# Placentation and fetal membrane development in the South American coati, *Nasua nasua* (Mammalia, Carnivora, Procyonidae)

**DOI:** 10.1186/1477-7827-12-57

**Published:** 2014-06-27

**Authors:** Phelipe O Favaron, João C Morini, Andrea M Mess, Maria A Miglino, Carlos E Ambrósio

**Affiliations:** 1 Department of Surgery, School of Veterinary Medicine and Animal Science, University of Sao Paulo, Av. Prof. Dr. Orlando Marques de Paiva, 87, Cidade Universitária, 05508-270 São Paulo, SP, Brazil; 2 Department of Veterinary Medicine, FZEA, University of São Paulo, Av. Duque de Caxias Norte, 225, ZMV, 13635-900 Pirassununga, SP, Brazil

**Keywords:** Carnivore, Endotheliochorial placenta, Hemophagous organ, Yolk sac

## Abstract

**Background:**

Placental research in carnivores has concentrated on domestic species, which have zonary, labyrinthine placentas with an endotheliochorial barrier. Although the coati, *Nasua nasua*, is a widely distributed species in South America, data on the development of the placenta and the fetal membranes in this species are very sparse.

**Findings:**

Four placentas from mid-gestation to near term were collected from wild individuals and were investigated based on gross morphology, histology, immunohistochemistry and electron microscopy. The available data support the concept that the ancestral condition of placentation in carnivores is phylogenetically characterized by a zonary and labyrinthine placental type with an endotheliochorial fetomaternal barrier, comprising extended epitheliochorial and haemochorial zones, such as hemophagous organs for iron supply and histiotrophe uptake and a yolk sac placenta.

**Conclusions:**

Because of the foundational mechanisms that lead to the considerable complexity of fetomaternal contact zones in carnivores have not been studied, carnivores are interesting animal models for interhaemal barrier differentiation.

## Findings

### Background

**Figure 1:**
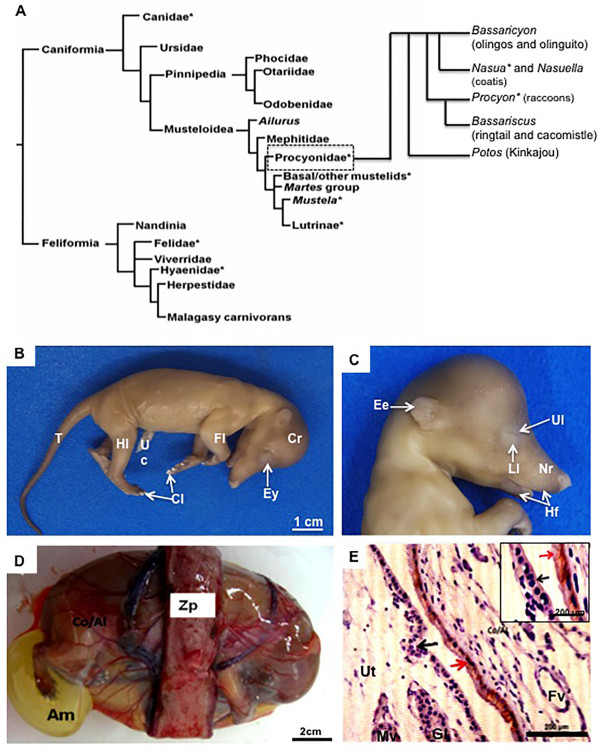
**Phylogeny of carnivores and features of the fetuses and placenta. (A)** Systematic relationships of Carnivora, with basal dichotomy between Caniformia and Feliformia (after [25,26]). The groups included in the comparison are marked by asterisks. **(B-C)** External features of the fetuses. Note the well-developed cranial region (Cr), forelimbs (Fl) and hindlimbs (Hl) with keratinized claws (Cl), and elongated tail (T). In the cranial region, note the eye (Ey) with upper (Ul) and lower (Ll) lips and pigmented retina. Hair follicles (Hf) were present in the elongated nasal region (Nr). Uc = umbilical cord. **(D)** Macroscopic anatomy of the term placenta in coati. Zp = zonary chorioallantoic placenta, Co/Al = noninvasive chorioallantois, Am = amnion. **(E)** Cytokeratin, mid-gestation. Chorioallantoic membrane (Co/Al) attached to the uterus (Ut), both with intact epithelia (arrows). Gl = uterine gland, Mv = maternal (arterial) vessel, Fv = fetal vessel.

Carnivores are regarded as having zonary, labyrinthine placentas with an endotheliochorial barrier. In addition, specialized areas are common, such as hematomas or hemophagous organs for the uptake of uterine secretion or the phagocytosis of extravasated maternal blood components, serving as mode of iron supply [1-3]. However, placental research in carnivores has mainly concentrated on domestic dogs and cats [4-11], rather than other species (e.g., [12-14]). Within Musteloidea, the mink, ferret and sea otter have been investigated [15-18], in addition to the raccoon and certain other species, including the coatimundi *Nasua narica*[19-21], (Figure 1A). Because more information, especially on wildlife species, is essential to better understanding the evolution of placentation in carnivores, we herein describe this system in the South American coati *Nasua nasua* (Linnaeus, 1766), which is native to southern South America, for the first time [22]. Coatis are omnivorous, medium-sized animals with a body mass of approximately 6 kg [21,22]. These animals live in groups organized within a complex social system consisting of up to 70 individuals. Two different groups are usually present; one composed entirely of adult males and a second group consisting of an alpha female (dominant), adult females, and young [23,24]. Females reach sexual maturity at 2 years of age, and males do so at approximately 3 years. The mating season usually occurs in late spring, from September to November. This is the only time when adult reproductive males meet with female groups [22-24]. After 10–11 weeks of gestation, 2–6 very altricial young with a body weight of 100 to 180 g are born [21,22]. Conclusions about the evolutionary course of the characters have been drawn from comparative analyses and analyzed within the relationships of carnivoran groups (Figure 1A), as indicated by recent morphological and molecular phylogenetics [25,26]. We use the terminology of phylogenetic systematics [27] in differentiating between derived character conditions and ancestral ones, with special reference to ancient conditions or the stem species patterns of Carnivora.

### Methods

The project was approved by the Bioethics Commission of the School of Veterinary Medicine and Animal Science, University of Sao Paulo, Brazil (Protocol Nr. 1598/2009), on July 1st, 2009, and by the Biodiversity Authorization and Information System – SISBIO (Protocol Nr. 21030–1) on September 17th, 2010. The study was performed from July 2009 to November 2011.

In total, four placentas from mid-gestation (n = 2) to near term (n = 2) were collected from 2 wildlife individuals captured in Mangabeiras Park in Belo Horizonte-MG, Brazil. Hemi-ovariohysterectomy was performed following surgical and anesthetic protocols used for domestic carnivores [28].

Measurements of the occipital-sacral distance of the fetuses’ heads were performed with a stainless-steel caliper, using the nuchal crest at one end and the last sacral vertebra at the opposite end as references (crown-rump, CR), following the methodology proposed by Evans and Sack [29]. The CR distances and the external features of the fetuses were used to estimate the age of each individual. The weight (g) was determined using a digital scale (0.0001 g; BEL Engineering).

After macroscopic examination of the main placental structures (zonary placenta, chorion, allantois, amnion, yolk sac, and hemophagous organs), the tissues were fixed in 4% paraformaldehyde for histology and immunohistochemical staining. The samples were stained for vimentin (1:400, 0.N.602, sc-73259, Santa Cruz Biotechnology, Santa Cruz, CA, USA) to identify mesenchymal cells and endothelium, for cytokeratin (1:300; M0821, Dako, Carpinteria, CA, USA) to identify epithelial and trophoblastic cells, and for proliferating cell nuclear antigen (PCNA; 1:300; PC10, sc-56, Santa Cruz Biotechnology, Santa Cruz, CA, USA) to identify proliferating cells. For this staining, we followed protocols established by our group [30]. The negative control was goat anti-mouse IgG (1:500; AP308F, Chemicon International Temecula, CA, USA). The slides were examined with an Olympus BX40 microscope with a Zeiss KS400 image analysis system. For scanning and transmission electron microscopy, placental samples were fixed in 2.5% glutaraldehyde and processed following established protocols [10,11].

### Results

#### Macroscopy of the fetuses

The two *Nasua nasua* offspring displayed CR lengths of 6.3 cm and 6.5 cm at mid-gestation and weighed 21.37 g and 23.88 g. Thus, their estimated age was 37–40 days of pregnancy. Near term (estimated age of 50–53 days of pregnancy), the offspring displayed CR lengths of 8.9 cm and 9.5 cm and weighed 33.61 g and 35.28 g. In both stages, the individuals had a body with distinct cranial, thoracic, and abdominal regions. An elongated and curved tail was also a feature observed in these stages of pregnancy. The forelimbs and hindlimbs were completely formed and presented keratinized claws on the fingers (Figure 1B). On the face, well-formed upper and lower lips were identified. The eyes displayed pigmented retinas. The nasal region was elongated, with several sensorial hair follicles, and the external ear was short but evident (Figure 1C). In addition, the entire body of the fetuses was covered by hair that was short, with dark pigmentation in certain regions, including the face, forelimbs and hindlimbs, and tail (Figure 1B,C).

#### The placenta and fetal membranes

The chorioallantoic placenta was fully zonary and surrounded each fetus in the abdominal region (Figure 1D). Outside the girdle, the trophoblast of the chorioallantoic membrane was apposed to the vascularized, gland-rich uterus, which had an intact, cubic epithelium (Figure 1E).

**Figure 2:**
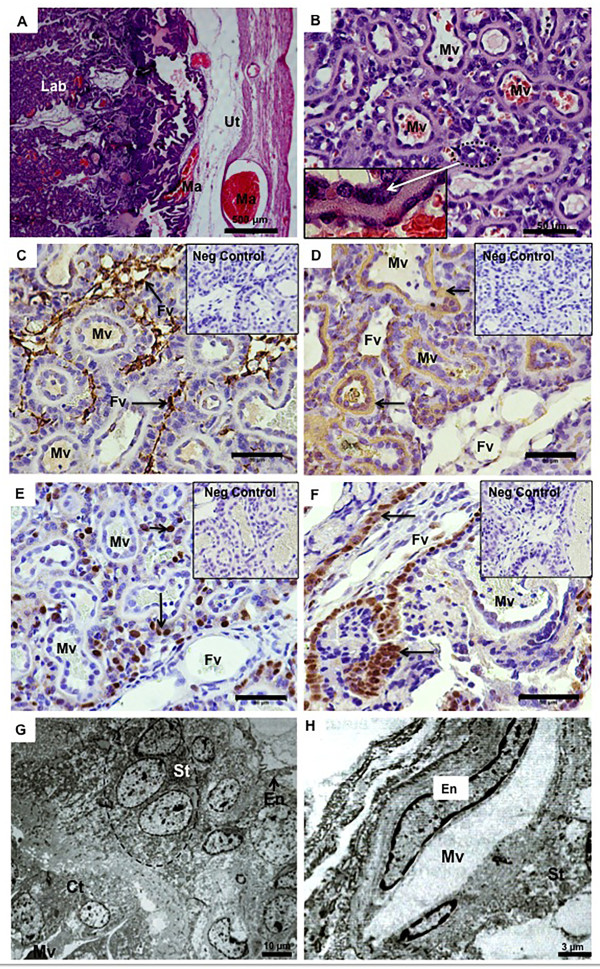
**Placental structure and fetal membranes. (A)** Hematoxylin and eosin, term placenta. Villi of the labyrinth (Lab), supplied by maternal arteries (Ma) from the uterus (Ut). **(B)** Hematoxylin and eosin, term. Detail of the maternal vessels (Mv) in the labyrinth and the cytotrophoblast clusters (circle and detail). **(C,D)** Immunohistochemical staining for vimentin and cytokeratin, respectively, near term. The mesenchyme of the fetal blood vessels (Fv) was strongly reactive for vimentin, in contrast to the maternal vessels (Mv). Cytokeratin in trophoblastic cells (arrows) near the maternal vessels (Mv). **(E-F)** PCNA, near term. In E: Proliferative trophoblastic cells (arrows) in the labyrinth, near the fetal vessels (Fv). Mv = maternal vessels. In F: Proliferation of the chorioallantoic branches (arrow) inside the labyrinth. **(G,H)** Transmission electron microscopy, mid-gestation. Cytotrophoblast (Ct) and syncytiotrophoblast (St) near a maternal vessel (Mv) and a fetal vessel with endothelium (En).

**Figure 3:**
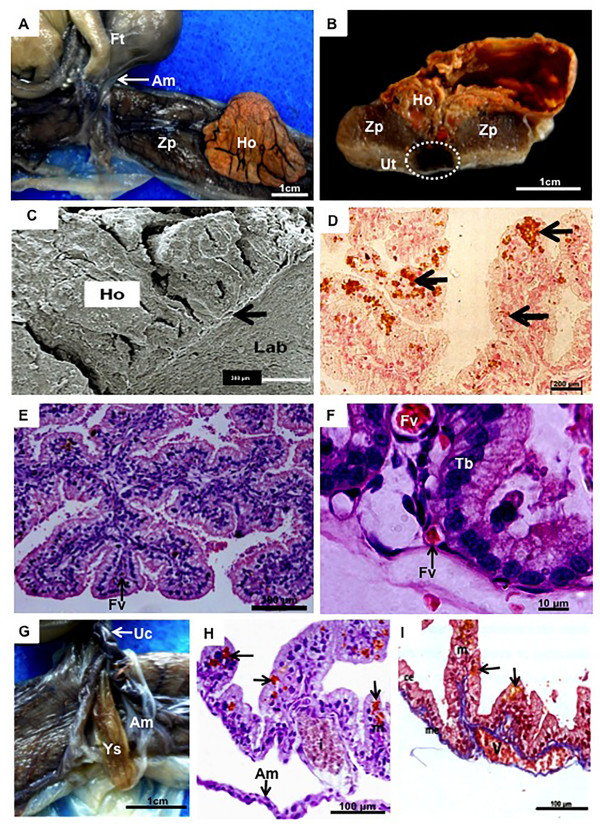
**Placental structure and fetal membranes. (A,B)** Macroscopy, near-term placenta. Sac-like hemophagous organ (Ho) central to the zonary placenta (Zp) and supplied by an area of destruction (circle) in the uterus (Ut). Am = amnion, ft = fetus. **(C)** Scanning electron microscopy, mid gestation hemophagous organ. The hemophagous organ (Ho) was near the labyrinth (Lab), separated by a thin membrane (arrow). **(D)** Pearl’s iron staining, mid-gestation. Iron deposits in the cytoplasm of the trophoblast (arrows). **(E,F)** Hematoxylin and eosin, near term. Villi of the hemophagous organ with a columnar trophoblast (Tb) and fetal vessels (Fv). **(G)** Macroscopy, term. The yolk sac (Ys) was surrounded by the amnion (Am). The umbilical cord (UC) was short. **(H)** Pearl’s iron staining, mid-gestation, with iron deposits in the yolk-sac endodermal cells (arrows). Am = amnion. **(I)** Masson’s trichrome, near term. Histology of the yolk sac, with mesothelium (me), mesoderm (m), and columnar endoderm (ce) with vacuoles (arrows).

The placental girdle in *Nasua nasua* had a prominent, lamellar labyrinth that was endotheliochorial in nature. Each lobe was supplied by central maternal and fetal vessels in a crosscurrent arrangement (Figure 2A,B). The mesenchyme of the fetal vessels was strongly reactive for vimentin, and it was possible to delimit the maternal and fetal blood systems in the labyrinthine zone (Figure 2C). In contrast, the trophoblast near the maternal vessels was positive for cytokeratin (Figure 2D). The trophoblastic cells close to the fetal blood vessels were especially proliferative, including in the branches of the chorioallantoic membrane near the placental girdle in mid-gestation (Figure 2E,F). Both cellular and syncytiotrophoblasts were frequent in mid-gestation (Figure 2G,H). Near term, the barrier was mainly syncytial and thin, but clustered cytotrophoblasts were present near the fetal vessels (Figure 2B). At that point, the endothelium of the maternal vessels was more hypertrophied than in mid-gestation (Figure 2H). In addition to the main placenta, the coati had a prominent, sac-like, orange area, vascularized by vessels from the umbilical cord that persisted until term (Figure 3A,B). Based on a centrally situated area of destruction allowing leakage of maternal blood, the area emanated into the allantoic cavity (Figure 3B) and was distinct from the labyrinth (Figure 3C). Iron deposits (Figure 3D) indicated a haemophagous nature. Inside, branched, vascularized villi were present, bathed in extravasated maternal blood (Figure 3E). The trophoblast was columnar, with large nuclei, apical vacuoles, and liquid droplets that stored ingested erythrocytes (Figure 3D,E).The amnion in the coati included large amounts of yellow-pigmented liquids (Figures 1D, 3G) and was avascular in mid-gestation but vascular near term due to fusion with the allantois (Figure 3H). The yolk sac was surrounded by the amniotic membrane (Figure 3G) and had a cubic epithelium composed of the endoderm, with iron deposits (Figure 3H,I). Numerous, large vitelline vessels were present, with blood cells forming vascular islets (Figure 3H,I).

### Discussion

Extended non-invasive, non-villous, fetomaternal contact zones similar to what is observed in epitheliochorial placentas are widespread in carnivores [4-21] and are known as the polar zone of the paraplacenta [7]. These zones likely uptake histiotrophe from endometrial glands [8] and represent an ancient character condition of the group Carnivora. The hyena is unique among carnivores because it presents a hemochorial villous placenta with intimate contact between the fetal and the maternal circulation via so-called intraepithelial capillaries in the syncytial trophoblast [13]. In contrast, true epitheliochorial placentas, serving as main fetomaternal contact zones, i.e., the complete reduction of invasive trophoblasts in maternal tissue interactions into strictly noninvasive forms, are regarded as evolutionarily derived conditions that have occurred in several clades of eutherian mammals, such as the ungulates and relatives [2,31-34]. Because there is little detailed information on the foundational processes of mammalian trophoblast invasion and its restriction, it is unclear whether the ontogenetic establishment of the carnivore paraplacenta follows similar processes as do the epitheliochorial placentas of close relatives within Laurasiatheria [2,32]. However, regarding the main fetomaternal exchange region of the placenta, comparative data [4-21] have confirmed that endotheliochorial, labyrinthine placentation belongs to the ancestral carnivore pattern [2,32]. Within the group, only hyenas have haemochorial placentas [13,14], which are hemomonochorial in nature, consisting of a continuous layer of syncytial trophoblasts, a basal lamina, and the fetal capillary endothelium [35].

Regarding the fine structure of the fetomaternal interface, the following characters are widespread among carnivores and seemed to belong to their stem species pattern or ancestral pattern: an endotheliochorial barrier in the zonary or circumferential placenta, in addition to a yolk sac and a large allantoic sac that persist until term. In contrast, the coati and other procyonids were derived from the ancient carnivore condition, maintaining the cellular trophoblast to term [19-21].

Hematomas (hemophagous organs), as specialized quasi-haemochorial zones in addition to the main endotheliochorial placenta, are widespread in carnivores. These structures are usually temporary, are mainly built by phagocytosing cytotrophoblasts [9,20] that are associated with uterine glands and extravasated blood, serve as the iron supply, and take up histiotrophe [4-21]. Thus, these organs represent multifunctional or heterophagous areas [1,3,14]. The ancestral pattern of carnivores likely had such areas at the placental margin, occurring in both Caniformia and Feliformia. *Nasua* and other Musteloidea [19-21] have large, multilobular organs in a central position. Moreover, *Nasua nasua* possesses a derived condition in that the organ persists, fully functional, until near term. Areolae are absent in the coati and in other Musteloidae, cats and hyenas [4,7,8,10,13-21] and likely are not part of the carnivore stem species pattern [2].

## Conclusions

The data on the coati supported previous views on the ancestral placental characters of carnivores; the maintenance of cellular trophoblasts in the barrier and the large, central hemophagous organ that persisted until near term were confirmed. The ancestral pattern of carnivores includes not only an endotheliochorial, labyrinthine placental girdle, but also extended epitheliochorial and hemochorial zones and placental function for the yolk sac. This considerable complexity of fetomaternal contact zones must have evolved before the radiation of carnivores, approximately 65 million years ago [25,26]. It is currently unclear what types of tissue-specific inflammation patterns and associated molecular mechanisms are involved in establishing specific fetomaternal contact in various regions. Thus, carnivores are interesting animal models for studying the full range of interhaemal barrier differentiation and function within an individual species. The coati may be of special interest to further study persisting hemophagous organs to better understand the nature and functional significance of substance transfer in the placental areas.

## Competing interests

There are no financial competing interests for any of the authors.

## Authors’ contributions

POF and JCM performed the practical analysis, advised by CEA. CEA and MAM devised the study and participated in its design. AMM and POF wrote the manuscript. CEA and MAM corrected the manuscript. All authors read and approved the final manuscript.

## References

[B1] Enders AC Carter AM Comparative placentation: some interesting modifications for histotrophic nutrition – a review Placenta 2006 27 Suppl 11 16 16406004 10.1016/j.placenta.2005.10.013

[B2] Mess A Carter AM Evolutionary transformations of fetal membrane characters in Eutheria with special reference to Afrotheria J Exp Zool B Mol Dev Evol 2006 306 140 163 16254985 10.1002/jez.b.21079

[B3] Enders AC Carter AM The evolving placenta: Convergent evolution of variations in the endotheliochorial relationship Placenta 2012 33 319 326 22364740 10.1016/j.placenta.2012.02.008

[B4] Amoroso EC Histology of the placenta Brit Med Bull 1961 17 81 90 13683045 10.1093/oxfordjournals.bmb.a069901

[B5] Anderson JW Ultrastructure of the placenta and fetal membranes of the dog. I - The placental labyrinth Anat Rec 1969 165 15 36 5806354 10.1002/ar.1091650103

[B6] Wynn RM Corbet JR Ultrastructure of the canine placenta and amnion Am J Obstet Gynecol 1969 103 878 887 5813064 10.1016/0002-9378(69)90588-2

[B7] Leiser R Enders AC Light- and electron-microscopic study of the near-term paraplacenta of the domestic cat Act Anat 1980 106 312 326 10.1159/000145195 7376812

[B8] Leiser R Koob B Development and characteristics of placentation in a carnivore, the domestic cat J Exp Zool 1993 266 642 656 8371103 10.1002/jez.1402660612

[B9] Stoffel MH Gille U Friess AE Scanning electron microscopy of the canine placenta Ital J Anat Embryol 1998 103 291 300 11315959

[B10] Miglino MA Ambrósio CE Martins DS Pfarrer C Leiser R The carnivore pregnancy: the development of the embryo and fetal membranes Theriogenology 2006 66 1699 1702 16563485 10.1016/j.theriogenology.2006.02.027

[B11] Ambrósio CE Brolio MP Martins DS Morini JC Carvalho AF Miglino MA Endometrial alterations, early placentation and maternal fetal interaction in carnivores Rev Bras Reprod Anim 2011 35 217 228

[B12] Michel G Elze K Seifert S Zur Embryonalentwicklung des Bären unter besonderer Beachtung des Baues der Plazenta Zoolog Gart 1983 53 290 294

[B13] Wynn RM Amoroso C Placentation in the spotted hyena (*Crocuta crocuta* Erxleben), with particular reference to the circulation Am J Anat 1964 115 327 362 14210298 10.1002/aja.1001150208

[B14] Enders AC Blankenship TN Conley AJ Jones CJP Structure of the midterm placenta of the spotted hyena, *Crocuta crocuta*, with emphasis on the diverse hemophagous regions Cells Tissues Organs 2006 183 141 155 17108685 10.1159/000095988

[B15] Enders AC Histological observations on the chorio-allantoic placenta of the mink Anat Rec 1957 127 231 245 13411590 10.1002/ar.1091270209

[B16] Sinha AA Mossmann HW Placentation of the sea otter Am J Anat 1968 119 521 554 5972735 10.1002/aja.1001190310

[B17] Krebs C Winter H Dantzer V Leiser R Vascular interrelationships of near-term mink placenta: light microscopy combined with scanning electron microscopy of corrosion casts Microsc Res Tech 1997 38 125 136 9260843 10.1002/(SICI)1097-0029(19970701/15)38:1/2<125::AID-JEMT13>3.0.CO;2-R

[B18] Pfarrer C Winther H Leiser R Dantzer V The development of the endotheliochorial mink placenta: light microscopy and scanning electron microscopical morphometry of maternal vascular cats Anat Embryol 1999 199 63 74 9924936 10.1007/s004290050210

[B19] Creed RFS Biggers JD Comparative placentation of the raccon Am J Anat 1963 113 417 446 14072366 10.1002/aja.1001130306

[B20] Creed RFS Biggers JD Placental haemophagous organs in the Procyonidae and Mustelidae J Reprod Fertil 1964 8 133 137 14195699 10.1530/jrf.0.0080133

[B21] Benirschke K Coatimundi, *Nasua narica yucatanica* 2014 http://placentation.ucsd.edu

[B22] Beisiegel BM Notes on the coati, *Nasua nasua* (Carnivora: Procyonidae) in Atlantic Forest area Rev Bras Biol 2001 61 689 692 10.1590/s1519-69842001000400020 12071327

[B23] Gompper ME Gittleman JL Wayne RK Genetic relatedness, coalitions and social behaviour of White-nosed coatis, *Nasua narica* Anim Behav 1997 53 781 787

[B24] Gompper ME Gittleman JL Wayne RK Dispersal, philopatry, and genetic relatedness in a social carnivore: comparing males and females Mol Ecol 1998 7 157 163 9532759 10.1046/j.1365-294x.1998.00325.x

[B25] Flynn JJ Finarelli JÁ Zehr S Hsu J Nedbal MA Molecular phylogeny of the carnivore (Mammalia): assessing the impact of increased sampling on resolving enigmatic relationships Syst Biol 2005 54 317 337 16012099 10.1080/10635150590923326

[B26] Nyakatura K Bininda-Emonds ORP Updating the evolutionary history of Carnivora (Mammalia): a new species-level supertree complete with divergence time estimates BMC Biol 2012 10 12 22369503 10.1186/1741-7007-10-12 PMC3307490

[B27] Henning W Phylogenetische systematic 1982 Berlin: Parey

[B28] Oliveira FS Toniollo GH Machado MRF Paura D Hemi-ovariossalpingo-histerectomia em pacas prenhes e posterior ocorrência de prenhez (*Agouti paca*, Linnaeus, 1766) Ciênc Rural 2003 33 547 551

[B29] Evans HE Sack WO Prenatal development of domestic and laboratory mammals: growth curves, external features and selected references Anat Histol Embryol 1973 2 11 45 10.1111/j.1439-0264.1973.tb00253.x 4745140

[B30] Favaron PO Carter AM Ambrosio CE Morini AC Mess AM Oliveira MF Miglino MA Placentation in Sigmodontinae: a rodent taxon native to South America Reprod Biol Endocrinol 2011 9 55 21518439 10.1186/1477-7827-9-55 PMC3094283

[B31] Mess A Carter AM Evolution of the placenta during the early radiation of placental mammals Comp Biochem Physiol A Mol Integr Physiol 2007 148 769 779 17347003 10.1016/j.cbpa.2007.01.029

[B32] Wildman DE Chen C Erez O Grossman LI Goodman M Romero R Evolution of the mammalian placenta revealed by phylogenetic analysis Proc Natl Acad Sci 2006 103 3203 3208 16492730 10.1073/pnas.0511344103 PMC1413940

[B33] Eliot MG Crespi BJ Placental invasiveness mediates the evolution of hybrid inviability in mammals Am Nat 2006 168 114 120 16874618 10.1086/505162

[B34] Capellini I Venditti C Barton RA Placentation and maternal investment in mammals Am Nat 2011 177 86 98 21087154 10.1086/657435

[B35] Oduor-Okelo D Neaves WB The chorioallantoic placenta of the spotted hyena (*Crocuta crocuta* Erxleben): an electron microscopic study Anat Rec 1982 204 215 222 7158827 10.1002/ar.1092040306

